# Reticulocyte and Erythrocyte Hemoglobin Parameters for Iron Deficiency and Anemia Diagnostics in Patient Blood Management. A Narrative Review

**DOI:** 10.3390/jcm10184250

**Published:** 2021-09-19

**Authors:** Christian Hoenemann, Norbert Ostendorf, Alexander Zarbock, Dietrich Doll, Olaf Hagemann, Mathias Zimmermann, Markus Luedi

**Affiliations:** 1 Marienhospital Vechta, Abteilung für Anästhesiologie und Operative Intensivmedizin, Marienstrasse 6–8, 49377 Vechta, Germany; dietrich.doll@kh-vec.de (D.D.); olaf.hagemann@kh-vec.de (O.H.); 2 St. Franziskus-Hospital Münster, 48145 Münster, Germany; norbert.ostendorf@sfh-muenster.de; 3 Department Westfälische, Wilhelms, Universität Münster, 48149 Münster, Germany; zarbock@uni-münster.de; 4 DRK Kliniken Berlin, Institut für Labormedizin, 14050 Berlin, Germany; m.zimmermann@drk-kliniken-berlin.de; 5 Department of Anaesthesiology, Inselspital, Bern University Hospital, University of Bern, 3012 Bern, Switzerland; markus.luedi2@insel.ch

**Keywords:** anemia, iron deficiency, patient blood management, reticulocyte hemoglobin (Ret He), anemia of inflammation (ACD, anemia of chronic decease), hospital acquired anemia

## Abstract

Anemia, iron deficiency and other hematinic deficiencies are a major cause of perioperative transfusion needs and are associated with increased morbidity and mortality. Anemia can be caused either by decreased production of hemoglobin or red blood cells or by increased consumption and blood loss. Decreased production can involve anything from erythropoietin or vitamin B12 insufficiency to absolute or functional lack of iron. Thus, to achieve the goal of patient blood management, anemia must be addressed by addressing its causes. The traditional parameters to diagnose anemia, despite offering elaborate options, are not ideally suited to giving a simple overview of the causes of anemia, e.g., iron status for erythropoiesis, especially during the acute phase of inflammation, acute blood loss or iron deficiency. Reticulocyte hemoglobin can thus help to uncover the cause of the anemia and to identify the main factors inhibiting erythropoiesis. Regardless of the cause of anemia, reticulocyte hemoglobin can also quickly track the success of therapy and, together with the regular full blood count it is measured alongside, help in clearing the patient for surgery.

## 1. Introduction

“Patient Blood Management” (PBM) and “Enhanced Recovery After Surgery” (ERAS) concepts are systematic quality improving clinical models. One major part is to diagnose and reduce anemia and avoid transfusions in all kinds of clinical settings. Here, we review the potential of Reticulocyte and Erythrocyte Hemoglobin Parameters for Iron Deficiency and Anemia Diagnostics in these more and more important clinical concepts.

The ERAS concept is a set of evidence-based practices which aim to optimize outcome, reduce patient surgical stress response, and improve recovery after surgery [1,2,3]. ERAS strives to optimize each patient’s condition, from preoperative, intraoperative and postoperative hospital intensive care treatment all the way to the post-discharge phase. This is achieved through patient education, preparation, and physiological optimization, including additional medical interventions as needed. It has been shown that even in the absence of full adherence, the closer practice is to ERAS standards, the fewer postoperative complications are observed and the shorter the hospitalization period needed [4,5]. When successfully implemented, ERAS can reduce morbidity by 50% and length of stay by 2.5 days, without an increase in readmissions [6]. This is not only beneficial for the patient, but also helps reduce costs and save resources, without cutting corners or putting patients at risk.

## 2. Patient Blood Management in the ERAS Concept

One important and modifiable factor affecting patients undergoing surgery is preoperative anemia. It affects more than a third of patients undergoing major surgery [7]. But even without manifest anemia, iron deficiency is an independent risk factor of worse outcomes [7]. Anemia, iron deficiency and other hematinic deficiencies are thus a major cause of transfusion needs and are associated with increased morbidity and mortality [7,8,9,10].

Iron deficiency is a major cause of preoperative anemia. But iron or other hematinic deficiencies need to be treated because they not only interfere with preoperative anemia therapy such as erythropoiesis stimulating agent (ESA) treatment, which is less effective when the iron supply is absent or insufficient, but also prevent postoperative recovery. Any blood loss because of surgery only exacerbates the problem, leading to postoperative anemia being even more common [11,12] and iron deficiency at all stages leads to a higher risk of complications.

As Anemia and iron deficiency are clinical problems that can be resolved prior to surgery, they are among the factors addressed by the ERAS concept. Patient blood management (PBM) is thus an important part of the ERAS concept and focuses on optimizing the care of patients who might need a blood transfusion [13].

The most immediate medical intervention used to counter anemia is the administration of packed red blood cells. However, use of blood products is associated with a substantial risk of complications and morbidity [14]. Consequently, patient blood management calls for tighter transfusion thresholds [15]. Transfusion itself, however, only prevents definitive diagnosis and management of anemia, again resulting in negative consequences for patients recovering from surgery. Thus, to achieve the goal of patient blood management, anemia has to be taken out of the equation by addressing its causes.

During a hospital stay, 25% of patients develop relevant anemia [16]. This is called Hospital-Acquired Anemia (HAA). It is very common that patients having normal hemoglobin values upon admission develop anemia over the course of hospitalization [17]. The incidence ranges from approximately 25–75% prior to discharge using the nadir hemoglobin values during hospitalization. Potential etiologies for HAA are iatrogenic blood loss from phlebotomy [18,19,20,21,22] or “anemia of chronic disease” induced by acute phase reactions stimulated by interleukins activating hepcidin synthesis [23,24,25]. HAA has been postulated to be a hazard of hospitalization that is potentially preventable [8]. Early diagnostic of anemia in critical care units is needed, to differentiate between HAA and other causes of anemia.

## 3. Causes of Anemia

Anemia is defined by the World Health Organization (WHO) as a decrease in hemoglobin concentration in the blood below certain sex- and age-specific levels—specifically <13 g/dL (8 mmol/L) for men, Hb < 12 g/dL (7.4 mmol/L) for women and <11 g/dL (6.8 mmol/L) for pregnant women [26]. There is, however, some discussion as to whether the statistically lower average concentrations of HGB in women indeed justify a distinct lower reference limited and do not in fact describe conditions that should better receive treatment.

Anemia can be caused either by decreased production of hemoglobin or red blood cells or by increased consumption and excessive blood loss or red blood cell destruction. Decreased of production can involve anything from erythropoietin or vitamin B12 insufficiency to absolute or functional iron deficiency.

### 3.1. Iron Deficiency Anemia

Iron deficiency is the most common cause of anemia [27], but reduced iron levels and low iron availability have multiple negative effects, and may be responsible for fatigue, organ dysfunction and other disorders [28,29]. Iron is not just a component of hemoglobin, but also of myoglobin, and thus, patients with severe heart failure are at particular risk [30,31].

If iron deficiency is caused, for example, by malnutrition, first the iron stores will be depleted, with all iron going directly to erythropoiesis, leading to low ferritin. As the iron deficiency worsens, transferrin saturation decreases as not enough iron can be mobilized to meet demands. Eventually, erythropoiesis will become iron deficient, with new reticulocytes not being provided enough hemoglobin (reduced reticulocyte hemoglobin). Finally, as more and more fully hemoglobinized red blood cells die off and are replaced with hypochromic ones, anemia fully manifests in a decrease in overall blood hemoglobin and microcytosis, until in the end the overall number of red blood cells decreases, too.

### 3.2. Anemia of Inflammation (ACD, Anemia of Chronic Disease)/Hospital-Acquired Anemia

However, certain conditions can block iron stores from releasing iron, leading to deficient erythropoiesis in the presence of filled iron stores. This can be found in patients with chronic disease, e.g., cancer, with rheumatic or other inflammatory diseases, or with chronic infections. While significant inflammation can also lead to suppression of erythropoietin production or sensitivity, in most cases, anemia of chronic disease or of inflammation is brought about via interference with iron metabolism. In these cases, pro-inflammatory cytokines are released, among which interleukin 6 leads to a subsequent release of hepcidin by the liver.

Hepcidin regulates iron metabolism by binding to the iron export channel ferroportin and causing its internalization [32]. Ferroportin is found both on the basolateral surface of enterocytes of the intestinal tract and in macrophages. Hepcidin thus blocks both the release of dietary iron into the hepatic portal system by the gut, and the uptake of dietary iron, as well as the release of iron stored in macrophages and thus the functional capacity of iron already stored in the body. During conditions in which the hepcidin level is abnormally high, such as inflammation, serum iron falls due to iron trapping within macrophages and liver cells and decreased intestinal iron absorption. This typically leads to functional iron deficiency due to an inadequate amount of serum iron being available for developing red blood cells. When the hepcidin level is abnormally low—as in hemochromatosis—iron overload occurs due to increased ferroportin- mediated iron efflux from storage and increased gut iron absorption [32]. The treatment of an iron deficiency will thus depend on the hepcidin levels that are present. Oral treatment will be unlikely to be effective if hepcidin is blocking enteral absorption, in which case parenteral iron treatment would be called for. Measuring hepcidin itself, however, is complex and not widely done. Instead, CRP is often used as a proxy to indicate the presence of inflammation.

### 3.3. Using Diagnostics to Differentiate between Iron Deficiency Anemia and Anemia of Chronic Diseases to Identify Adequate Treatment

The complex differential diagnostics of iron deficiency has led to a host of different parameters. Among the parameters traditionally used to assess iron deficiency are the soluble iron in the blood, the iron in ferritin storage, transferrin, and transferrin saturation. These have substantial disadvantages in terms of reliability, especially during an acute phase reaction and in the presence of anemia due to chronic diseases (ACD) (Table 1). On the other hand, due to the lifetime of red blood cells (~120 days), parameters measuring directly characteristics of the bulk of red blood cells and overall hemoglobin have a large delay in their response.

Serum iron has high variability between and within individuals and is neither sensitive nor specific. Ferritin is generally seen as the gold standard for iron deficiency but is in fact specific for iron storage deficiencies: While a low value indicates a lack of storage iron, ferritin is an acute phase reactant, and a normal or even high value can mask insufficient availability of iron for erythropoiesis. [33] (p. 1) Transferrin illustrates the demand for iron but can be depressed in chronic inflammation. The same holds true for its saturation. The soluble transferrin receptor or the sTfR-Fe index are likewise subject to acute phase reaction to some degree but are particularly problematic in that they are not well standardized and expensive, and as such not available in some smaller hospitals. Thus, the traditional parameters, despite offering elaborate options, are not ideally suited to giving a reliable overview of the iron status for erythropoiesis, especially during the acute phase. An alternative was thus needed.

## 4. Reticulocyte Hemoglobin

Reticulocyte hemoglobin (measured as RET-H_e_ or CHr, proprietary names of the manufacturers of haematology analyzers Sysmex (Kobe, Japan) and Siemens Healthineers (Erlangen, Germany), respectively) [34,35] is a measurement of the mean hemoglobin contained in reticulocytes, the immature red blood cells freshly released from the bone marrow. RET-H_e_ (reticulocyte hemoglobin equivalent) is derived from forward scatter of the reticulocyte population identified in the optical RBC scattergram. These are the cells with the highest side fluorescence obtained by a nucleic acid stain. As reticulocytes are the direct products of erythropoiesis, they offer an almost immediate snapshot of the availability of iron, and as they mature within a few days, they are not drowned out by older cells produced during a different iron status as is the case for mature red blood cells. The reticulocyte count itself is a good parameter for the quantitative aspect of erythropoiesis, but hemoglobinization of reticulocytes gives additional information about quality of erythropoiesis directly reflecting the hemoglobinization. RET-H_e_ can thus detect a latent subclinical iron deficiency even before the manifestation of full iron deficiency anemia [36,37]. As it directly reflects the erythropoiesis, the parameter is not confounded by acute phase reaction [38,39]. It responds quickly (within about 2 days), is accurate [40], and is inexpensive, as it is part of a blood count from specific proprietary hematology analyzers. This makes it a much more practical and available solution for measuring iron availability to erythropoiesis than the reference method involving bone marrow aspiration and Prussian-Blue staining. The two main parameters of reticulocyte hemoglobin measurement have been shown to be highly correlated and concordant [35,41] and can be used independently of the specific solution mentioned in guidelines.

While it has long been known that anemia is associated with the need for blood transfusion and greater mortality in surgical patients, it has more recently been shown that the same holds true for critically ill patients with low mean reticulocyte hemoglobin (<29 pg) [42].

But what role can reticulocyte hemoglobin play in anemia diagnostics?

**Figure 1:**
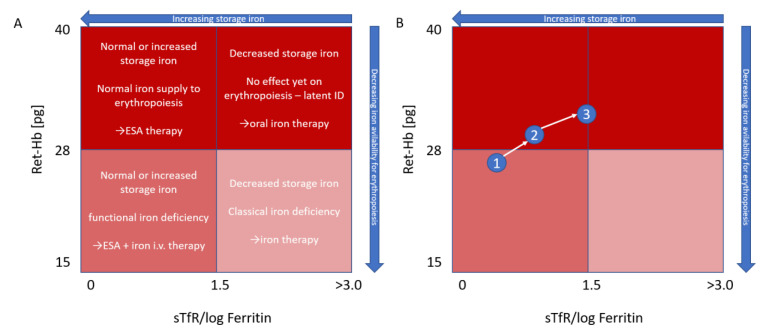
Modified Thomas plot [38]. Characterization (**A**) and monitoring (**B**) of anemia based on reticulocyte hemoglobin and the sTfR/log ferritin index. In (**B**), an initial functional iron deficiency (1) is first treated with ESA and i.v. iron therapy (2). Subsequent focus on ESA therapy leads to reduction of storage iron to a borderline level, while hemoglobinization of reticulocytes is still normal (3), Ret-Hb = Ret-H_e_ = mean reticulocyte hemoglobin.

The use of reticulocyte hemoglobin, possibly plotted against an index calculated from sTfR/log ferritin (Figure 1), if other clinical chemistry parameters are desired despite the drawbacks mentioned, was proposed by Thomas & Thomas [38] as far back as 2002, with the goals being to distinguish between absolute and functional iron deficiency and to track iron repletion. It thus stands to reason that reticulocyte hemoglobin should also be able to play a role as a more accurate, sensitive, timely and inexpensive option to know which anemia to treat with iron.

## 5. Treatment

In patients with renal anemia, especially those undergoing hemodialysis, although the cause of anemia is generally an EPO deficiency, secondary ACD can still lead to iron-restricted erythropoiesis and interfere with therapy. Ferritin values can be normal or elevated, even when no iron is available. Reticulocyte hemoglobin measurement can show that iron is functionally deficient and not arriving in the bone marrow [33,43]. In this case, guidelines then recommend intravenous iron for dialysis patients. Reticulocyte hemoglobin can subsequently be used to track recovery of the iron supplied to erythropoiesis (Table 2) [43,44].

Anemia of chronic disease is the second most common cause of anemia [45]. As in patients undergoing dialysis, chronic inflammation or malignant diseases can cause functional iron deficiency. Iron will again be unavailable, with the unwanted side effect of depriving the bone marrow of iron, too.

Reticulocyte hemoglobin has been shown to be a reliable diagnostic marker even under these conditions that iron supply is limited for the bone marrow for erythropoiesis [46] and can track the response to therapy. Its use, alone or in the form of the plot mentioned above, has been suggested or advised in reviews and recommendations for the diagnosis and management of anemia of chronic disease [47] as well as for determining the usefulness of iron administration.

In the case of absolute iron deficiency, ferritin and reticulocyte hemoglobin can be expected to both be low. As iron demand is high and there is no present need to store iron, reticulocyte hemoglobin can track the response to iron repletion therapy more directly.

Reticulocyte hemoglobin can thus help to uncover the cause of the anemia and to identify the main factors inhibiting erythropoiesis [48]. Regardless of the cause of the anemia, reticulocyte hemoglobin can quickly track the success of therapy and, together with the regular full blood count it is measured alongside with, clear the patient for surgery.

Depending on the cause of the anemia, simple oral iron supplementation may be sufficient if surgery is not urgent. In patients with preexisting chronic inflammatory conditions, i.v. iron may be necessary.

## 6. Postoperative Iron Deficiency

Diagnosis of postoperative iron deficiency can be even more difficult than that of preoperative deficiency since ferritin levels may be elevated as part of the acute phase response after surgery [49]. As a result, RET-H_e_ is recommended as a reliable parameter to assess iron availability in the management of patients with postoperative anemia after major surgical procedures [11]. Even in acute surgical patients, removing any bottlenecks can help prevent major postoperative complications, and giving iron one day before or even during surgery has been shown to improve postoperative erythropoiesis [50,51]. Therapeutic interventions to counteract the anemic condition should be started immediately upon classification and RET-H_e_ can help show whether iron or ESA are needed. In some cases of severe anemia, especially in the presence of comorbidities, transfusions may of course still be necessary. For iron treatment, intravenous iron is generally indicated, as an inflammatory response to the surgery is likely to prevent the availability of iron stores postoperatively.

The use of reticulocyte hemoglobin can provide an inexpensive snapshot of the status of erythropoiesis, ensuring that even latent iron deficiency is recognized before surgery. Measures to mitigate the iron deficiency can then be implemented in a timely fashion, thereby also reducing the risk of adverse events due to RBC concentrate treatment during surgery.

## 7. Patient Blood Management (PBM) and Iron Deficiency

For Germany alone, modelling suggests that in the context of iron deficiency anemia, implementation of rigorous preoperative anemia management could save €536 million in direct costs and a further €503 million through reduced length of stay of elective surgery patients, for a total of more than €1000 million, at the same time avoiding 3036 hospital deaths. Meanwhile, the costs of implementing preoperative anemia management were estimated to be €10–17 million, depending on the iron dosage used [52].

However, the preoperative management of anemia in the context of Patient Blood Management is currently practiced in a very heterogeneous way, even in countries with highly developed healthcare systems [53]. Lack of knowledge and awareness about the impact on outcomes of anemia and iron deficiency, and the complicated pathway to assess and treat anemia, may be a contributing factor. Reticulocyte hemoglobin content or equivalent, on the other hand, could be incorporated into routine preoperative evaluation with a regular blood count, without requiring additional blood samples and only minimal added costs, providing invaluable information on most causes of anemia, and facilitating management. It therefore has the potential to become a fixed parameter in PBM and ERAS.

## 8. Potential Role of Ret-He in Treatment of Iron Deficiency in Septic Patients

**Figure 2:**
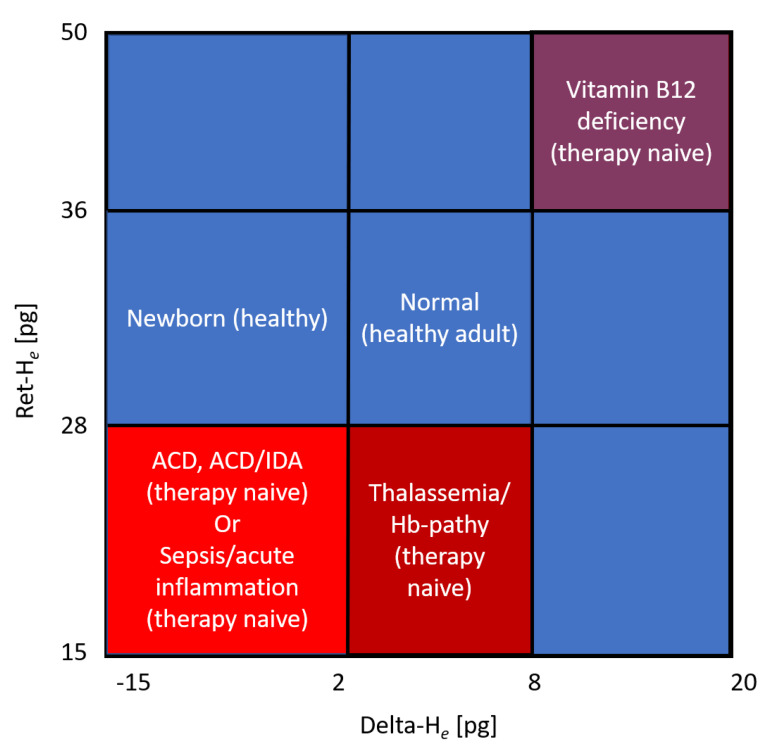
Hema-plot (adapted from Weimann et al. [56]) Characterization of anemia based on reticulocyte hemoglobin (RET-H_e_) and the difference between it and the hemoglobinization of mature erythrocytes (Delta-H_e_).

Iron is a key factor in microbial growth and the downregulation of iron uptake and transport by hepcidin thought to be part of an immune response [54]. Consequently, there are some who oppose iron treatment in sepsis patients for fear of facilitating microbial growth, preferring to use packed red blood cells when anemia becomes critical. However, not only are some bacteria not affected by this type of iron restriction, but some are also capable of using heme as a source of iron [55], making transfusion in that aspect functionally similar to iron therapy. Moreover, erythrocyte concentrate therapy is independently associated with a significant risk of complications and mortality. The question arises as to whether reticulocyte hemoglobin, perhaps in association with other parameters, could help fine-tune iron treatment so that it meets the needs of the bone marrow but avoids fostering bacterial growth. Tracking iron replenishment in erythropoiesis by comparing the hemoglobinization of the mature erythrocytes, measured through the same technology as that of the reticulocytes, may indicate when iron therapy can be stopped, but also illustrate that iron is being sequestered in the first place. The corresponding parameter for this difference has been dubbed Delta-H_e_ by Sysmex. It is calculated by subtracting RBC-H_e_ from RET-H_e_. RBC-H_e_ is the haemoglobin content of erythrocytes and therefore roughly comparable to MCH but measured with the same technique as RET-H_e_. Together with reticulocyte hemoglobin, it can also be used to obtain a much more differentiated assessment of anemia, as certain constellations are characteristic for different underlying causes [56] (Figure 2).

## 9. Outlook

Combining these reticulocyte-related parameters with a more complex characterization of white blood cells may provide a better idea as to what type of pathogen is causing the inflammation. Variations in side scatter and fluorescence of white blood cells can reflect the activation and reactivity of lymphocytes, monocytes or neutrophils. This would provide peace of mind when it comes to starting i.v. iron. For the time being, the proposed Intensive Care Infection Score (ICIS) can help differentiate bacterial infection from non-infectious systemic inflammation [57]. The ICIS comprised five blood-cell derived parameters that characterize the early innate immune response: (I) mean fluorescence intensity of mature (segmented) neutrophils; (II) difference in haemoglobin concentration between newly formed and mature red blood cells; (III) total segmented neutrophil count; (IV) antibody secreting lymphocyte count; (V) immature granulocyte count [57].

**Figure 3:**
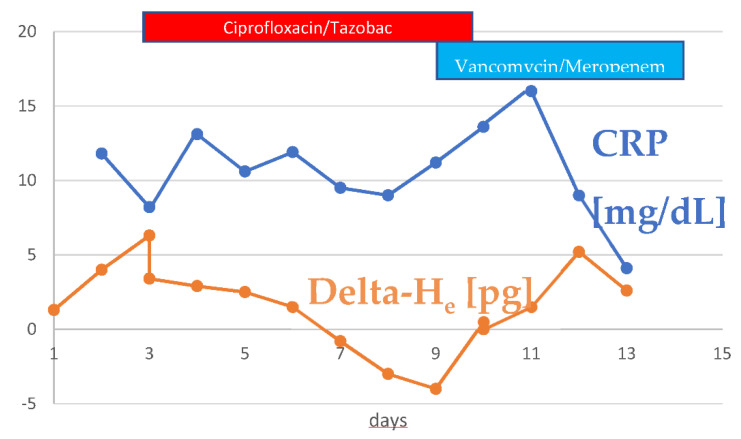
Example for Delta-He in a sepsis patient. The response to a change in antibiotic therapy is already apparent in the increase in Delta-He after 12 h—Other parameters respond much more slowly.

Initial studies of the ICIS that include the parameter Delta-H_e_ have been promising [57,58,59]. The difference between hemoglobinization of mature erythrocytes and reticulocytes is important in this context (Figure 3).

**Figure 4:**
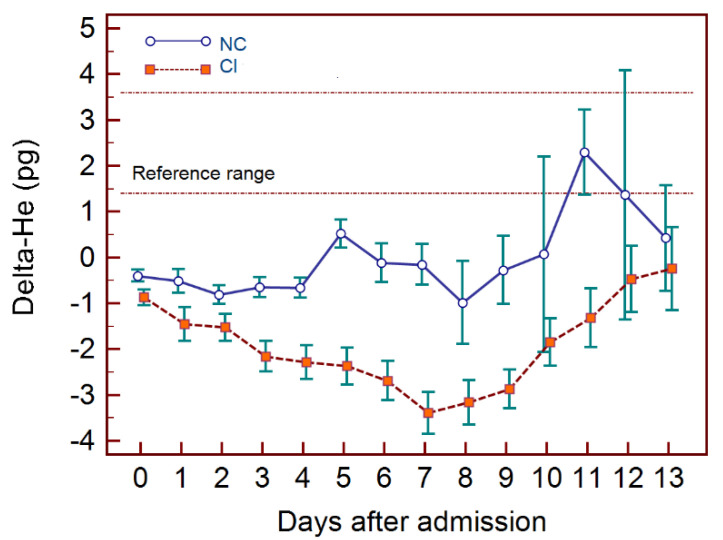
Delta-He in COVID-19 patients. Difference between critical (CI) and non-critical patients (NC).

In fact—and this leads us to the current pandemic—a similar score also including RET-H_e_ and Delta-H_e_ has recently been proposed as a prognostic tool in COVID-19 infections [60]. The aim of this study was to develop a prognostic score (based on multiple hematocytometric parameters) to predict during the first three days after presentation, which patients will recover without ventilation or deteriorate within a two-week time frame. Here, too, Delta-H_e_ has an important contributing role in score composition, showing markedly different behavior in critical vs. non-critical patients (Figure 4).

As for Patient Blood Management, already in 2006 orthopedic surgeons and clinical chemists in the Netherlands published a protocol optimizing the treatment of both pre- and postoperative anemia by detecting iron deficiency using RET-He [61]. Over the course of the previous years, this protocol had allowed them to reduce the annual number of packed red blood cells needed for major hip and knee surgeries by 83% through the strategic administration of iron sucrose in patients in whom RET-H_e_ indicated iron deficiency.

More recently, preoperative anemia walk-in clinics have been established at several hospitals in Germany. In one of them, use of RET-H_e_ was tested and eventually adopted as a routine parameter to screen for anemia before orthopedic surgery [62]. Including a score to detect infection generated from the same hematology analyzers in such protocols may, in the end, also resolve concerns about iron therapy in cases where severe infection may be suspected.

## Figures and Tables

**Table 1:** Overview of iron deficiency parameters.

Parameter	Elevated	Reduced	Shows	Disadvantages
Serum Iron	Iron overload (preanalytical hemolysis)	Iron deficiency and acute/chronic inflammation	Amount of iron (Iron bound to transferrin & non-transferrin bound iron)	High intra- and inter-individual variability Neither sensitive nor specific
Ferritin	Acute phase, liver disease, lymphoma	Iron deficiency with depleted iron storage	Iron stores	Acute phase reactant, no direct conclusion for erythropoiesis
Transferrin	Iron deficiency, pregnancy (last trimester)	Chronic inflammation, tumors, hemolysis	Transport iron, demand by erythropoiesis	By itself only indicates demand, not supply
Transferrin saturation	Iron overload	Iron deficiency, acute phase, pregnancy (last trimester)	Percentage of filled transferrin binding sites	Acute phase reactant, requires two measurements (transferrin, iron)
Soluble transferrin receptor	Increased erythropoiesis during iron deficiency	Chronic kidney disease with reduced EPO	Secreted fragment of transferrin receptor	Not elevated during acute phase, reference interval highly dependent on specific test, expensive
Transferrin receptor	Iron overload	Iron deficiency, acute phase, pregnancy (last trimester)	Target of transferrin iron transport, mediates iron uptake by endocytosis	Acute phase reactant
sTfR-Ferritin index	Iron deficiency	Anemia of chronic disease	sTfR/log Ferritin	Requires two measurements, thus complex and costly
Hepcidin	Iron deficiency anemia, CKD, inflammation	Iron overload	Iron absorption and release from storage	Complex measuring methods, reference interval highly dependent on specific technology
RET-H_e_		Iron deficient erythropoiesis	Functional availability of iron	Not available from all manufacturers

CKD = chronic kidney disease; EPO = erythropoietin; sTfR = soluble transferrin receptor; Ret-H_e_ = mean reticulocyte hemoglobin.

**Table 2:** Evaluation of iron deficiency in chronic kidney disease.

Cellular assessment	Hb < 11 g/dL RBC indices (MCH, MCHC, MCV) White blood cell and differential count Platelet and reticulocyte count
Iron assessment	Hypochromic cells % (if sample < 6 h old) Reticulocyte Hb (RET-He) Serum ferritin C-reactive protein
Iron therapy targets	Hypochromic cells < 6% Reticulocyte Hb (RET-He) > 29 pg Ferritin > 100 µg/L TSAT > 20%

Hb = mean hemoglobin, RBC = red blood cells, MCH = mean corpuscular hemoglobin, MCHC = mean corpuscular hemoglobin concentration, MCV = mean corpuscular volume, Reticulocyte Hb = Ret-H_e_ = mean reticulocyte hemoglobin, TSAT = transferrin saturation.
